# Cultural Differences in Answerability Judgments

**DOI:** 10.3389/fpsyg.2018.01641

**Published:** 2018-09-18

**Authors:** Bodil S. A. Karlsson, Carl Martin Allwood

**Affiliations:** Department of Psychology, University of Gothenburg, Gothenburg, Sweden

**Keywords:** answerability, judgment, answerability judgments, knowledge questions, confidence judgments, cross-cultural comparison

## Abstract

Judgments about whether anyone can provide a relevant and correct answer to a question are called *answerability judgments*. Such judgements can be important in societal planning and decision making and may vary in different cultural contexts. Six hundred participants in each of China, India, and Sweden made answerability judgments of six difficult knowledge questions. For each question, they choose between three options indicating that they thought the question was answerable and a fourth option: “*Nobody can answer that question.*” After each question, they rated their confidence that their judgment was correct. Choosing “*Nobody can answer that question*” was significantly more common for the Swedes and was uncommon in the Asian samples. The Asian samples showed higher confidence in their judgments. We suggest that these differences may be explained by results from cross-cultural research, but this study did not investigate specific mechanisms. Hence, more research is needed.

## Introduction

The correct answers to important questions like “is there a safe way to store nuclear waste?,” “will the polar-ices melt in 500 years?,” and “is radiation from cell-phones dangerous?” have been debated in politics and within science. In such debates, people may even deliberate if anyone can provide a correct answer, that is, if the question is *answerable.* It can be difficult to judge if questions relating to complex issues have been answered. Furthermore, when questions are seen as unanswered but possible to answer, it can be difficult to judge how much work this will take.

Answerability judgments of questions of the type exemplified are important since they may affect other subsequent important decisions, e.g., policies about nuclear energy. In general, answerability judgments are likely to be influenced by conditions that relate to the person’s prior experiences due to socialization, upbringing and education, etc. In brief, cultural differences in understanding, cognitive approach, and cues heeded from the social context can lead to differences between countries in the judged answerability of questions. If answerability judgments vary cross-culturally, this would be useful to know in international negotiations and collaborations.

It is therefore important to investigate such judgments across cultural variations. Apart from the theoretical interest of this issue, it also holds practical interest since realistic assessments of questions’ answerability are important in many social contexts such as safety (e.g., new building materials; chemicals in medicine, food, clothing and toys; citizen security; new technology), medical or crime investigations, effects of social reforms and new laws, and in the context of existential and religious issues. Moreover, in an increasingly global world, people from different cultures have to interact and try to collaborate in order to find solutions for common challenges involving complex issues such as those exemplified above (Savani et al., 2015).

The present study investigated answerability judgments in three cultural contexts, China, India, and Sweden, of complex fact-oriented questions concerning health and sustainability related issues. It also investigated confidence judgments of these judgments in the same three countries. The three countries were selected in order to get a spread in cultural conditions that may influence answerability judgments. As described below, research has indicated that the three countries tend to differ in their thinking style.

It should be noted that we do not claim to investigate *actual answerability* and the study does not deal with philosophical problems about truth and knowledge. By measuring judgments of answerability we are trying to tap a person’s *belief* that “a correct, well-argued, answer at a relevant granularity level can be provided to the question” (Allwood et al., 2016, p. 40). Answerability judgments can be more or less correct, but their correctness may, at the time of making the judgment, be unknown to the person who makes the judgment, often due to the complex nature of the problem.

### Previous Research on Answerability Judgments

Basic research on answerability judgments is scarce and needed. Allwood et al. (2016), Karlsson et al. (2016), and Buratti et al. (2017) explored answerability judgments of knowledge questions with controversial, and less controversial, answers in a Swedish context. Karlsson et al. (2016) studied answerability of perceptual questions. Different answerability scales were successfully used in this research. Karlsson et al. (2016) collected answerability judgments from two groups of participants made on two different answerability scales, *the future scale* and *the current scale*, and then compared the level of the judgments for each of 22 questions on the two scales by calculating a correlation between judgments on the two scales over all questions. The authors reported that a group of participants’ ratings of the distance in time of when a question could be answered (*the future scale*: now, in a year, in two years, in ten years, etc., ...or never) correlated strongly with another group of participants’ ratings of how probable it was that someone could answer the same questions today (*the current scale*). This result indicates some robustness of different forms of answerability scales. Buratti et al. (2017) investigated answerability judgments that related to questions’ answerability *today* with a categorical answerability scale where the alternatives were “I know,” “someone else knows,” and “no one knows.” These answer alternatives are similar to those used in the present study.

### Cross-Cultural Research

Much research shows that judgments are influenced by the socially prevalent understanding in the cultural context the judger lives in (e.g., Perkins, 1993; Cole, 1996; Atran et al., 2005; Koriat, 2008, 2012; Sloman and Rabb, 2016). Moreover, people’s socialization provides them with a mental approach or thinking style related to their training experiences (Cole, 1996; Nisbett et al., 2001). Different types of cultural differences may affect answerability judgments.

A well-known, although somewhat debated, finding in cross-cultural psychology is that societies tend to differ in the extent to which they can be characterized as *collectivistic* or *individualistic* (Triandis, 1994; Oyserman et al., 2002; Varnum et al., 2010). Oyserman et al. (2002) presented a meta-analysis of cross-national studies and studies within the United States and found clear evidence that PRC Chinese and, to a slightly lesser extent, Indians are more collectivistic and less individualistic than Euro-Americans in the United States and people from Northern Europe, including the countries closest to Sweden, Finland, and Norway (Sweden was not included in the Oyserman et al. study). Similarly, Hofstede’s Individualism index show that China, India, and Sweden score 80, 77, and 31, respectively, indicating higher individualism for Sweden (Clearlycultural.com, 2018a,b).

Although obviously a matter of degree and with individuals showing variation within a society, people in collectivistic societies tend to see themselves as more part of, and dependent on collectives, including so-called interdependent self-construal, and people in individualistic societies tend to see themselves as independent individuals and less dependent on in-groups, including so-called independent self-construal (see research reviewed in, e.g., Varnum et al., 2010). In line with this, Hsee and Weber (1999) reported that Chinese people had greater social networks than the participants from the United States. It should also be noted that the distinction between collectivistic and individualistic societies has been criticized (e.g., Fiske, 2002; Miller, 2002). Poortinga (2016) warned of too simplistic interpretations of the collectivism/individualism and in this context cited a study by Sinha and Tripathi from 1994 which found indications of both collectivism and individualism in India. In spite of this, the collectivistic/individualistic distinction may still reasonably be seen as informative to some extent.

There is by now strong evidence supporting that social orientation (collectivism/individualism) may be an important cause of the prevalence of a specific thinking style in a society (Varnum et al., 2008, 2010). For example, Varnum et al. (2010), in a research review on this issue, noted that different studies show that thinking style and type of social orientation co-vary across and within cultures. Moreover, a meta-analysis by Oyserman and Lee (2008), described by Varnum et al. (2010), shows that the priming of either collective or of individualistic social orientation caused increased tendency to use the thinking style associated with the primed social orientation (see also Savani et al., 2015).

More specifically, the collectivism/individualism distinction has been linked to a difference in thinking style described as holistic and analytic thinking. People in collectivistic societies such as China tend to show a more holistic thinking style compared to societies in the West, and people in the West, including Sweden, tend to show a more analytic style. The holistic thinking style is more contextual and broad and the analytic style is more focused and abstract (e.g., Nisbett et al., 2001; Nisbett, 2003; Gutchess and Indeck, 2009). Moreover, people with a more holistic thinking style have a broader pattern of attention and attend to more relations between heeded entities. In line with this, they also attend more to context and background. In contrast, people with an analytic style have a more narrow association span and attend more to central elements (e.g., Varnum et al., 2008). For example, research reviewed in Ji et al. (2000) (see also Savani et al., 2015) shows that Asians tend to explain the behavior of other people in terms of contextual and situational factors. Given the research reviewed above, a more holistic thinking style may lead to a broader range of associations, for example when pondering answerability judgments.

Other cross-cultural differences may also influence answerability judgments. Research has found that Chinese people tend to have a longer time perspective compared with Western people. As reported by Hofstede and Minkov (2010), of the three countries represented in this study, China has been found to have the longest time perspective (value 118), and Sweden the shortest (33) with India (61) in between. In line with this, research has also found Chinese and participants from other Asian countries to be more patient (i.e., less discounting of the future) than Western people (Chen et al., 2005; Savani et al., 2015). In addition, on a general note, it deserves to be mentioned that research indicates that people in India show similarities in their thinking to people in East Asia (Savani et al., 2015). Though thinking styles have been cross-culturally compared, to our knowledge, no prior research has studied answerability judgments in a cross-cultural context and the present study thus contributes by being the first to do so.

### Confidence

Research on the accuracy of confidence judgments has found that people in China and other countries in Asia, including India but not Japan, show more overconfidence than Westerners, specifically the United States (Wright et al., 1978; Wright and Phillips, 1980; Yates et al., 2002; Yates, 2010; but see Lundeberg et al., 2000). This has also been found to be the case in research that controlled for the effect of proportion correct answers (Yates et al., 1989). In line with Chinese people showing higher confidence judgments, Ji et al. (2000) found that Chinese people, compared to United States people, gave higher confidence judgments of their covariation judgments.

Confidence judgments were also measured in this study. However, these were made after each answerability judgment. Therefore, the confidence judgments may not have influenced the answerability judgments. In line with the tendency in previous cross-cultural research on confidence judgments, we expected higher confidence ratings of the correctness of their answerability judgments in the Asian samples compared to the Swedish sample.

### The Present Study

The present study investigated answerability judgments of questions in China, India, and Sweden. We used the Internet to ask participants to judge the answerability of six complex fact-oriented questions. On the basis of reasoning and the research reviewed above, we expected that participants from China and to some extent those from India (together “Asians”) would make fewer “*nobody can answer that question*” judgments than the Swedes (Hypothesis 1). One reason for this hypothesis is that Asians may to a higher extent believe in collective efficacy, the possibility that people together can accomplish goals (Klassen, 2004) and socioinstrumental control (Spector et al., 2004). Furthermore, Asians’ purportedly higher tendency to more holistic thinking (broader range of associations) compared to Westerners (e.g., Swedes) may facilitate for them to construct more possibilities to answer the answerability judged question and by this making it more likely that someone may know the answer.

Finally, given that previous research has indicated that Asians tend to be more overconfident (Wright et al., 1978; Wright and Phillips, 1980; Yates et al., 2002; Yates, 2010), we expected Asians to be more confident in their answerability judgments (Hypothesis 2). Since our main research question related to answerability judgments, it is also of interest to investigate the participants’ confidence in their judgments.

## Materials and Methods

### Participants

Data were collected in a web-survey until 600 complete^1^ answers were acquired from each of China, India, and Sweden (52.7% men 46.5% women). **Table 1** shows the background characteristics for the participants from each country. As shown in **Table 1**, the mean age was highest in China, education level was highest in the Indian sample, and the proportion females was highest in Sweden. Mean age differed significantly between countries [*F*(2,1770) = 100.21, *p* < 0.001; Bonferroni correction *p* < 0.001]. Participants from India had significantly higher education level than participants from China and Sweden [*F*(2,1796) = 16.30, *p* < 0.001; Bonferroni correction *p* < 0.001], but the Swedish sample and Chinese sample did not differ significantly from each other on education level. Pearson Chi-square tests showed that the percentage women differed significantly between all countries (*p* < 0.001).

**Table 1:** Age (mean and range), education level (% participants at university level), and gender for the Chinese, Indian, and Swedish participants (*n* = 600 in each country).

	China	India	Sweden
Age (mean)	34.6	27.4	29.5
Age range	(17–59)	(16–80)	(17–80)
Education level (% university level)	90%	96%	86.3%
Gender	F = 292, M = 306	F = 112, M = 485	F = 433, M = 157
	Other = 2	Other = 1, no answer = 2	Other = 10

### Ethics

The present study followed ethical guidelines in Sweden for survey data. The Chinese participants were recruited via a Swedish marketing research firm and participants in India were recruited via Mturk. Participants in Sweden were recruited from a pool of adults that had already actively volunteered and signed up for participation in psychological research and can thus be considered consciously aware of participation in general.

Participants were recruited from participant panels of adults that had actively signed up to participate in online research. Participants received written information online about the study, that they had the right to end their participation at any time, that participation was anonymous, and that the data would be treated confidentially and only for research purposes. They were also provided with relevant contact information in case they had questions. They gave their consent and agreed to participation by clicking on the survey link.

### Materials

#### Judgments of Knowledge Questions’ Answerability

We used six knowledge questions concerning complex phenomena such as health, new technology, and environmental pollution. The six questions were: *“is radiation from cell phones dangerous?,” “will the polar ices melt in 500 years?,” “is there today a safe way to store nuclear waste?,” “does the human body have an unknown system of circulation?,” “are new electric cars more environmentally friendly than ordinary cars?,” and “is too much stress a greater danger to humanity than overweight?”* The first four questions have previously been rated low on answerability in Swedish samples (Allwood et al., 2016; Karlsson et al., 2016; Buratti et al., 2017).

For each of the six knowledge questions, there were four response options *(“yes”; “no”; “I don’t know, but I am sure somebody else knows”; “nobody can answer that question”).* The first three options were interpreted as indicating that the participant thought the question was answerable. After choosing one of the four response options, participants were asked to judge how confident they were that their answer was correct on a scale ranging from *“0%, I am guessing”* to *“100%, I am completely sure”* in steps of ten. After confidence judging their answer, participants clicked on “next” in order to see the next knowledge question. The six pages, each with a knowledge question and a confidence judgment, were presented in a randomized order. It was not possible to go back to questions on previous pages.

The languages used were: Chinese in China, English in India, and Swedish in Sweden. Necessary translations were made with back-translation procedure (Brislin, 1970).

#### Additional Question Material

Other questions were also asked. More detailed information about these questions can be found in the **Supplementary Material** and is, in the interest of brevity, not be reported in this manuscript.

### Procedure

Data were collected with a web-survey in China, India, and Sweden. After agreeing to participate in the web-survey, participants performed answerability judgments of the six knowledge questions and after each question rated their confidence in that the answerability judgment was correct. The participants also answered questions about age, gender, and education level.

## Results

### Answerability Judgments

**FIGURE 1:**
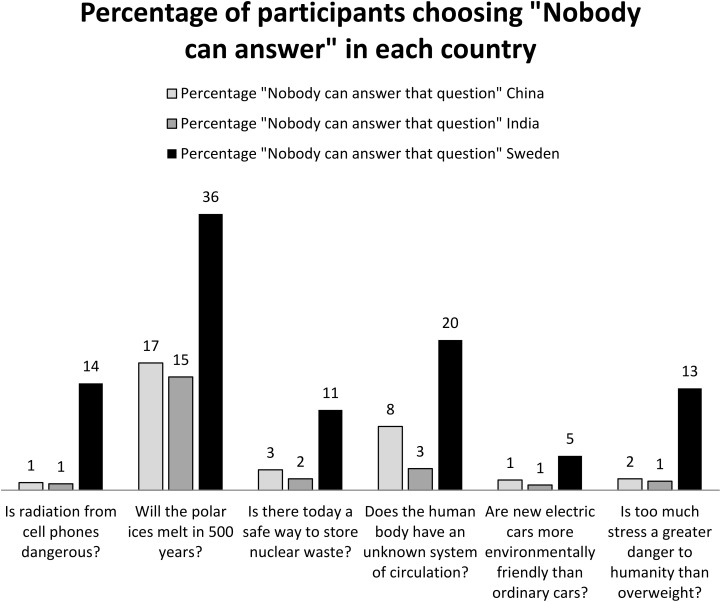
Belief in China, India and Sweden that the question is not possible to answer today. The numbers refer to the percentage (%) of the *n* = 600 participants in each country that chose the alternative “*Nobody can answer that question*” for each of the six answerability questions in each of the three countries.

Choosing the alternative “*Nobody can answer that question*” was interpreted as the question was judged not to be answerable. The other answer alternatives were used in order to make the answerability judgment task natural for the participants. However, our main topic of interest was to measure the prevalence of the belief that the question was not possible to answer by anyone (i.e., the number of “*nobody can answer that question*” judgments). As can be seen in **Figure 1**, Sweden scored significantly higher on prevalence of “*nobody can answer that question*” compared to both China and India on all questions (*p* < 0.05). Chi-square statistics ranged between χ^2^ = 12.04 for the smallest difference in prevalence of *“nobody can answer that question”* (the difference between Sweden and China for the question about electric cars) and χ^2^ = 71.87 for the largest difference in prevalence of *“nobody can answer that question”* (the difference between Sweden and India for the question about the polar ice). Taken together these results strongly support our expectation in Hypothesis 1 that Swedes would be more prone to choose *“nobody can answer that question.”*

### Confidence Judgments

The Swedish sample provided significantly lower confidence judgments of their answerability judgments for each of the questions compared to the Asian countries, except for the question about a nuclear waste. The method of analysis was a multivariate ANOVA with the six confidence judgments as dependent variable and country as independent, *F*(12,3586) = 30.78, *p* < 0.001. *Post hoc* analysis was made with Bonferroni correction (*p* < 0.05). The result provided support for Hypothesis 2, that Asians would show more confidence in their answerability judgments than the Swedes.

## Discussion

The purpose of this study was to explore how answerability judgments of difficult knowledge questions (e.g., “will the polar-ices melt within 500 years”?) differed between Chinese, Indian, and Swedish respondents with respect to the number of choices of the alternative “*nobody can answer that question.*” This is to our knowledge the first study investigating cultural differences in answerability judgments. In line with Hypothesis 1, the Swedish sample significantly more often chose *“nobody can answer that question”* for each of the six answerability judgments. Choice of this alternative was uncommon among the Asian respondents. Below we discuss some factors that may have contributed to these results.

### Cultural Differences in Belief in Collective Efficacy and Thinking Style

We compared two Asian countries (China and India) with a Western country (Sweden). Although this study did not investigate the specific mechanisms associated with the answerability judgments, results from research in cross-cultural psychology may help explain why the Asian samples gave fewer *“nobody can answer that question”* responses in their answerability judgments. With respect to belief in collective efficacy, collectivistic countries such as China have been found to show higher belief in this kind of efficacy, that is that people can accomplish goals together (Klassen, 2004; see also Spector et al., 2004), and this may have increased the Asian respondents’ tendency to think that someone has the answer to the judged question. Klassen noted that Bandura (1997) argued that collective efficacy should not be seen as replacing self-efficacy but should be seen as an addition to it, thus higher belief in collective efficacy can reasonably be expected to increase the perceived probability that at least someone in the collective knows the answer.

Turning next to thinking style, previous research supports that the Asian samples are more likely to have used a holistic thinking style (e.g., Nisbett et al., 2001; Nisbett, 2003; Gutchess and Indeck, 2009). Such differences in thinking style may influence answerability judgments. For example, as noted in the Introduction, the holistic thinking style is associated with attending to a broader range of associations, compared to the analytic thinking style that probably was more represented in the Swedish sample. A broader range of associations, combined with a general tendency for people to actively construct meaning in order to understand and create a meaningful world, rather than to de-construct their understanding of the world (e.g., Bartlett, 1932; Nickerson, 1998) is likely to allow for seeing more possibilities that the question answerability judged can be answered. Thus, such a broader holistic thinking style may make it easier to construct possible ways that the answer can be answered. If more ways to answer a question can be envisioned, the question may be more likely to be seen as possible to answer.

### Face-Saving Strategies, Uncertainty Avoidance, and Other Possible Contributing Factors

In addition, other factors may have contributed to the Asian samples finding the questions to be more answerable. For example, differences in cultural values such as sensitivity to other people’s opinions, and face-saving strategies may help explain why the Asian samples were more convinced that somebody could answer the question. Merkin et al. (2014) found that uncertainty avoidance (which is likely to include avoiding saying “*nobody can answer that question*”) is positively related to both sensitivity and face-saving concerns. In addition, the Asian schooling system is suggested to be more authoritarian than the Western system (Shu et al., 2010). Such differences in training might also possibly foster cultural differences in beliefs that questions usually are possible to answer. Finally, research results show that Chinese people and other Asians may have a longer time perspective and more patience. Karlsson et al.’s (2016) finding showing that there was a correlation between *the future* and *the current* answerability scales (measuring perceived answerability today and answerability today or in the future, respectively) indicates that judgments using these two scales may be driven by the same sort of factors, although this issue is not sufficiently researched. In brief, longer time perspective and greater patience may contribute to a higher general belief that questions are answerable but may not necessarily have contributed to the Asian respondents thinking that questions are answered today.

In general, as noted, the data from this study do not provide direct evidence that the factors suggested as contributing to our results actually caused our findings and further research should investigate the specific mechanisms associated with answerability judgments in the respective countries. Thus, further research should measure these various aspects directly and relate them to the participants’ answerability judgments.

### Confidence Judgments

Finally, in line with our expectations, we found the Chinese and Indian samples to be significantly more confident, compared to the Swedish sample on all questions, except one. Thus, our participants evidenced similar results for their confidence in their answerability judgments as previous research has found for confidence in the correctness of direct answers to, for example, general knowledge questions (Wright et al., 1978; Wright and Phillips, 1980; Yates et al., 2002; Yates, 2010). Accordingly, given that our results replicate in future research, people from east and south Asia might feel more convinced of their judgments about questions answerability in two ways; they may to a higher extent believe that questions are in fact answerable and they may also be more emphatic about these judgments given that they are more confident about them.

### Limitations and Practical Implications

Like all research this study has a number of limitations. Some of these are, first, that this was a questionnaire study and the results may not be fully representative of answerability judgments made in real life contexts. Therefore, future research should attempt to study such judgments in contexts that are more similar to every-day contexts, for example, by asking for answerability judgments in the context of more elaborated vignettes, or, even better, but more difficult, study them in real life. Second, our study was conducted on the net and the results, like in much cross-cultural research, are likely to be most representative for well-educated people in urban contexts. Third, our results only pertain to the answerability judgments of six questions. Although these questions were constructed with the aim to be about complex, socially relevant issues, it is still possible that our results primarily are representative for the content domains covered in the questions. Finally, we cannot exclude that there are also other differences in the samples unrelated to culture that matter for answerability judgments.

In spite of these limitations, our results highlight the importance that people who participate in cross-cultural collaborations within, or between, countries are aware of the possibility that people with different cultural backgrounds may well judge the extent to which important issues are possible to answer differently. Our results suggest that people from east and south Asia may to a higher extent believe that questions are in fact answerable and they may back up their judgments with different levels of confidence. Such differences may also sometimes influence east and south Asians, compared with Westerners, to collectively be more prepared to take on complex issues to the extent that they may judge the difficult questions relating to such issues to be more answerable.

## Author Contributions

All authors listed have made a substantial, direct and intellectual contribution to the work, and approved it for publication.

## Conflict of Interest Statement

The authors declare that the research was conducted in the absence of any commercial or financial relationships that could be construed as a potential conflict of interest.

## Footnotes

1 With complete we mean that participants had answered all the six answerability judgments.
