# Supplementary material for: Cultural Differences in Answerability Judgments
Source: Front Psychol. 2018 Sep 18;9:1641. doi: 10.3389/fpsyg.2018.01641 (PMC6153327; doi:10.3389/fpsyg.2018.01641)
Supplement: Supplementary file 1 [file Data_Sheet_1.docx]

Materials

Judgments of knowledge questions’ answerability. We used six knowledge questions concerning complex phenomena such as health, new technology and environmental pollution. The six questions were: *“Is radiation from cell phones dangerous?”, “Will the polar ices melt in 500 years?”, “Is there today a safe way to store nuclear waste?”, “Does the human body have an unknown system of circulation?”, “Are new electric cars more environmentally friendly than ordinary cars?”, “Is too much stress a greater danger to humanity than overweight?”*  The first four questions have previously been rated low on answerability in Swedish samples (Allwood et al., 2016; Buratti et al., 2017; Karlsson et al., 2016).

For each of the six knowledge questions there were four response options *(”yes”; “no”; I don’t know, but I am sure somebody else knows”; “Nobody can answer that question”).* After choosing one of the response options, participants were asked to judge how confident they were that their answer was correct on a scale ranging from *“0%, I am guessing”* to *“100%, I am completely sure”* in steps of ten. After confidence judging the answer, participants clicked on “next” in order to see the next knowledge question. The six pages, each with a knowledge question and a confidence judgment, were presented in a randomized order. It was not possible to go back to questions on previous pages.

Judgments of colors. We asked two sets of questions regarding the color judgments. The first set concerned The Jacket (Figure 1) and the second set concerned The Dress (Figure 2). Since The Dress was likely to be more well-known as having ambiguous colors than The Jacket, we presented The Jacket questions before The Dress questions in order not to raise suspicion that the photograph of The Jacket was ambiguous.

***Figure 1***. The Jacket, is a viral phenomenon that was spread due to individual differences in color perception.

**
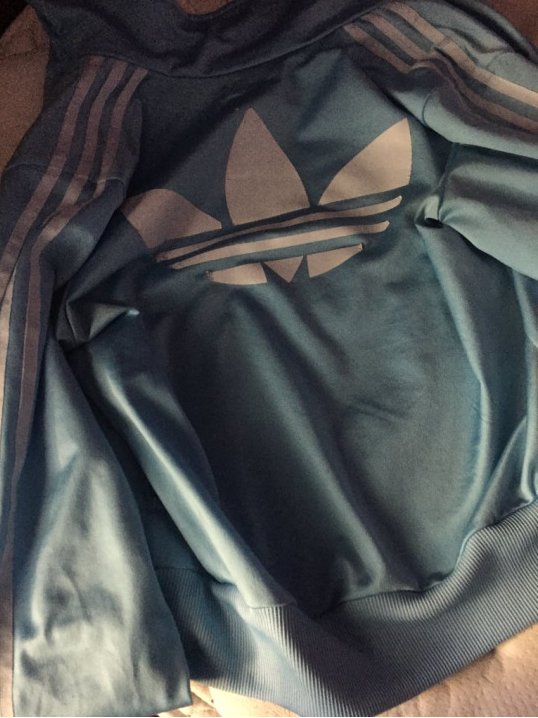
**

***Figure 2.*** The Dress: Blue or black or white and gold? The viral phenomenon #TheDress has received much scientific attention due to differences in color perception (e.g., Wallisch, 2017).

**
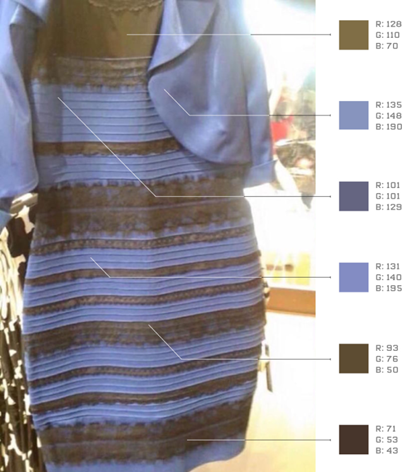
**

The questions about The Jacket color were presented in the following order. *“Please look at the photograph above* (A photograph of The Jacket was displayed above this question)*. “Have you seen this photograph of the jacket before?”* (*“yes”; “no”; “don’t know”*). *“How would you describe the colors you perceive the jacket to have?”* (*“white and blue”; “green and gold”; “other, namely,…”*).

Then there was a page-break and the photograph of The Jacket in question was no longer visible. The next question was: *“What is the correct answer about the colors of the jacket?”(*“*white and blue”; “green and gold”; other namely…”; “I don’t know, but I am sure somebody else knows”; “Nobody can answer that question”*). Below the question about the correct answer there was a confidence judgment: *“How confident are you that your answer is correct?”* The response was rated on a scale ranging from “0%, I am guessing” to “100%, I am completely certain” in steps of ten*.*

For those who claimed they had seen The Jacket before, an extra question was then displayed on a new page: *“What colors did you see the first time you saw the dress photograph?” (“white and blue”; “green and gold”; “other, namely…”, “I don’t remember”)* It was not possible to go back to questions on previous pages.

The same set of questions were then asked for The Dress, but the words *“the jacket”* were replaced with *“the dress”*  and the suggested color options *“white and blue”* and *“green and gold”* for The Jacket were replaced with “*blue and black”* and *“white and gold”* for The Dress.

Optimism. Individual optimism was measured with LOT-r which has six items (Monzani, Steca, & Greco, 2014). An example of an item is “*In uncertain times, I usually expect the best*”. The items were rated on a five-point scale ranging from 1 = *“Do not agree”* to 5 = *“Totally agree”.*  A Chinese translation was made with back-translation. The Swedish translation of the scale by Muhonen and Torkelson (2005) was used. Cronbach’s alpha was .64.

**Locus of control.** Locus of control was measured with Sapp and Harrod’s (1993) short version of Levenson’s locus of control scale. The scale has nine items and the items were answered on a five point scale ranging from 1 = *“Do not agree”* to 5 = *“Totally agree”.* The nine items are in turn divided into three subscales (own actions, chance and powerful others). Examples of an item from each subscale are: *“I am usually able to protect my personal interests”* (own actions), *“To a great extent my life is controlled by accidental happenings”* (chance), *“I feel like what happens in my life is mostly determined by powerful people”* (powerful others). Cronbach’s alpha were for Own Actions: .66, Chance: .61 and Powerful Others: .80.

**Circadian type.** Participants answered a measure of Circadian typology in line with Adan & Almirall, 1990) In this self-report measure participants answered 5 questions concerning among others, what time they usually go to bed, get up in the morning, and if to what extent they considered themselves being morningpersons/eveningpersons. The result was scored according to Adan & Almirall (1990) instructions, resulting in a first raw score between 0 and 25. Scores 22-25 means strong morningperson, 18-21 means morningperson, 12-17 means neither, 8-11 means evening person and 4-7 means strong eveningness. In analyses we used the raw score in order not to lose variance.

The languages used were: Chinese in China, English in India and Swedish in Sweden. Necessary translations were made with back-translation procedure (Brislin, 1970).

**Procedure**

Data was collected with a web-survey in China, India and Sweden. After agreeing to participate in the web-survey, participants were randomized to one of two order conditions. Approximately half of the participants started with judgments of knowledge questions and then performed the set of color judgments. The other half made the judgments of colors first and the knowledge questions second. After finishing the blocks with color judgments and knowledge questions, questions about age, gender and education level was answered. Last in the session participants answered the LOT-r -scale on personal optimism, the Circadian type scale.
